# A systematic review of diabetes self‐management education interventions for people with type 2 diabetes mellitus in the Asian Western Pacific (AWP) region

**DOI:** 10.1002/nop2.340

**Published:** 2019-09-03

**Authors:** Arbaktun Mohamed, Emily Staite, Khalida Ismail, Kirsty Winkley

**Affiliations:** ^1^ Department of Psychological Medicine Institute of Psychiatry, Psychology and Neuroscience King’s College London London UK; ^2^ Florence Nightingale Faculty of Nursing Midwifery & Palliative Care King’s College London London UK

**Keywords:** Asian Western Pacific region, diabetes self‐management education, interventions, systematic review, type 2 diabetes mellitus

## Abstract

**Aims and objectives:**

To assess the effectiveness of educational and/or psychological diabetes self‐management education (DSME) intervention for people with type 2 diabetes (T2DM) in the Asian Western Pacific (AWP) region.

**Background:**

Translational research indicates that DSME is effective; therefore, it is important to look at the AWP region to see what has been implemented and what the potential barriers are for the low integration of DSME. The need for DSME is present, and programmes are being developed. Therefore, focusing a systematic review of DSME research in the AWP region would give a better understanding of which intervention approaches are associated with better clinical outcomes and are culturally acceptable.

**Design:**

A systematic review.

**Methods:**

A review of randomized controlled trials (RCTs) and comparative studies to evaluate the effectiveness of face‐to‐face delivery reporting educational and/or psychological interventions for people with T2DM was implemented. We conducted searches using MEDLINE, EMBASE, CINAHL, PubMed and ASSIA databases between January 1990–June 2018. Studies published in English and non‐English were included. Two reviewers independently extracted data on participant and intervention characteristics. The quality of evidence was rated on predetermined criteria. Main outcomes included glycaemic control (reduction in HbA1c level).

**Results:**

We included 21 DSME programmes (17 RCTs), while 15 were group‐based approaches. Twelve studies (60%) were categorized as high quality. Three studies (25%) had a moderate (good) effect. Eight trials were effective in improving glycaemic control and reported statistically significant improvements in HbA1c levels. 50% of these were high‐intensity group‐based programmes.

## 1 INTRODUCTION

Diabetes mellitus (DM) is a highly prevalent chronic disease associated with serious and costly complications largely the result of obesity and physical inactivity. Around 387 million people live with DM worldwide, and type 2 diabetes mellitus (T2DM) is the most common comprising 90% of those with diabetes (American Diabetes Association, 2004). Furthermore, 138 million people with T2DM live in the Asian Western Pacific (AWP) region representing 30% of the total number of people with diabetes around the world (International Diabetes Federation, 2013). The AWP region encompasses East Asia (China, Japan, Republic of Korea), South‐East Asia (SEA) (Indonesia, Malaysia, Singapore, Brunei, Thailand, Myanmar, Laos, Cambodia, Vietnam, Philippines) and Oceania (Australia, New Zealand and French Polynesia in the East). It is diverse in terms of ethnicity, politics, economy, health systems, resources, cultural and religious beliefs. As a result of urbanization, access to cheap processed food and sedentary lifestyles, more people living in the AWP region are obese (prevalence 8.6%) (Foliaki & Pearce, 2003) leading to an increased prevalence of insulin resistance and T2DM. Furthermore, genotype studies suggest that risk factors for DM are different in the AWP population compared with Caucasians, such as genetic differences and altered fat distribution (Gao, Salim, Lee, Tai, & van Dam, 2012).

## 2 BACKGROUND

Diabetes self‐management education (DSME) provides information and skills needed by people with T2DM to effectively self‐manage their diabetes, in addition to medical management, to achieve optimum glycaemic control (Funnell & Anderson, 2004). Developed countries such as the United Kingdom (UK) and United States of America (USA) have successfully integrated DSME into their health systems, both have national guidelines for DSME provision (Haas et al., 2012; NICE Internal Clinical Guidelines Team, 2015). In Western cultures, DSME is designed by the guiding principles: (a) it is effective for improving clinical outcomes and quality of life; (b) it evolves from theoretically based empowerment models; (c) although there is no “best” educational approach, culturally and age appropriate programmes incorporating behavioural and psychosocial strategies demonstrate improved outcomes; (d) ongoing support is critical to sustain progress and; (e) behavioural goal setting is an effective strategy (Funnell et al., 2008). Programmes such as diabetes education and self‐management for ongoing and newly diagnosed (DESMOND) are associated with improved clinical outcomes (Davies et al., 2008), and the Diabetes Self‐Management Program (DSMP) demonstrated improvement in depression, healthy eating habits, more effective patient–health provider relationship, communication and self‐efficacy (Lorig, Ritter, Villa, & Armas, 2009) in the UK and USA, respectively. Meanwhile, also in the USA, the Diabetes Empowerment Education Program (DEEP) for Latinos reported significant improvement in glycaemic control (Castillo et al., 2010).

In 2014, the International Diabetes Federation's (IDF) global diabetes scorecard reported that less than 1% of countries in AWP region integrate DSME in their health services (International Diabetes Federation, 2013). Previous research suggests that DSME is effective; therefore, it is important to apply these findings to the AWP region, taking into account what has been implemented previously, as well as looking at what the potential reasons are for this low integration of DSME. The need for DSME is present, and programmes are being developed. Therefore, focusing a systematic review of DSME research in the AWP region would give a better understanding of which intervention approaches are associated with better clinical outcomes. The aim of this review was to synthesize the evidence for DSME programmes employing educational and/or psychological interventions for people with T2DM in the AWP region, looking at whether culturally specific techniques or sessions are incorporated. The results from this study may direct the local health providers to develop tailored DSME programmes to suit the diverse local population.

## 3 METHODS

This systematic review did not require patient consent or Research Ethics Committee approval.

### 3.1 Search strategy

Following PRISMA guidelines, eligible studies were identified from MEDLINE and EMBASE using the Ovid platform; CINAHL in the EBSCOhost platform; PubMed; and Web of Science and ASSIA from the ProQuest platform. The searches used the PICO (P: patient or problems; I: intervention being considered; C: comparison intervention; O: outcome measurements) framework (Davies, 2011) and were performed on 7 August 2015 (updated on 16 August 2015 and 21 June 2018). Table 1 demonstrates the search strategy and keywords used (“diabetes mellitus” and “diabetes education”). Exploded keywords were included and MESH terms for MEDLINE and modified truncation according to the different search platforms.

**Table 1:** Detailed search strategies for the systematic review of Diabetes Self‐Management Education (DSME) interventions for people with type 2 diabetes mellitus (T2DM) in the Asian Western Pacific (AWP) region

Search	String
1	Type 2 diabetes mellitus.mp. or exp non‐insulin dependent diabetes mellitus
2	Diabetes mellitus.mp. or exp diabetes mellitus/
3	#1 OR #2 Interventions Terms
4	Health education.mp. or health education/
5	Diabetes education.mp. or exp diabetes education/ or exp patient education/ or exp self‐care/
6	Diabetes self‐management.mp.
7	Exp behaviour therapy/ or behv$ therapy.mp.
8	behav$ intervention.mp.
9	psych$ intervention.mp.
10	Exp psychotherapy/ or psych$ therapy.mp.
11	#4 OR #5 OR #6 OR #7 OR #8 OR #9 OR #10
12	(American Samoa or Australia or Brunei Darussalam or Cambodia or China or Cook Islands or Fiji or French Polynesia or Guam or Hong Kong or Japan or Kiribati or Macao or Malaysia or New Caledonia or New Zealand or Niue or Northern Mariana Islands or Palau or Papua New Guinea or Philippines or Republic of Korea or Samoa or Singapore or Solomon Islands or Thailand or Tonga or Tuvalu or Marshall Islands or Micronesia or Mongolia or Nauru or Vietnam or Vanuatu or Wallis).mp. [mp = abstract, heading word, drug trade name, original title, device manufacturer, drug manufacturer, device trade name, keyword]
13	#3 AND #11 AND 12

Note

This search strategy was developed for EMBASE and modified to correspond the terminology for other databases.

### 3.2 Selection criteria

This systematic review included comparative studies. This is defined as RCTs, non‐RCTs and observational studies that used a comparison group. The broad inclusion criteria ensured all studies measuring effectiveness of DSME in adults aged 18 and over with T2DM in different healthcare settings were included. No limit was made on the language of publication. Eligible papers that were written in a different language were translated. Studies where participants were diagnosed with type 1 diabetes mellitus (T1DM), gestational diabetes and a mixture of T2DM with T1DM or other chronic conditions were excluded. Furthermore, studies solely investigating pharmacological or medication adherence, diet, exercise, physical activity, web‐based or peer support or telephone counselling were excluded to reduce confounding bias (Inzucchi et al., 2012). Only studies that covered more than one component of diabetes self‐management were included. Besides, only studies implemented exercising face‐to‐face delivery approach were included as discussed in a recent review regarding effectiveness of intervention using information technology and it suggested they were not as effective as a face‐to‐face method (Pillay et al., 2015).

The American Association of Diabetes Educators (AADE) defines DSME as “the ongoing process of facilitating the knowledge, skill and ability necessary for prediabetes and diabetes self‐care” (Haas et al., 2012, p.620). The DSME programme employs a patient‐centred approach as it helps people with T2DM to change their behaviour and achieve seven specific self‐care behaviours: healthy eating, being active, monitoring, taking medication, problem‐solving, healthy coping and reducing risks. DSME interventions were classified into (a) being predominantly educational interventions; or (b) predominantly psychological interventions (which include mental health). Educational interventions were defined as those that provide information on diabetes, its causes and management (medications or self‐management) (Verkuijlen, Verhaak, Nelen, Wilkinson, & Farquhar, 2014) and may include didactic and facilitative teaching approaches. Didactic teaching is a traditional lecture‐based teaching methodology that is teacher centred, while facilitative teaching refers to a learner‐centred approach and is more flexible combining teaching methodology with practical sessions such as exercise classes and telephone follow‐ups (Prince & Felder, 2006). In addition, the facilitative teaching approach may be underpinned by behavioural theory whilst the intervention is being developed (Jackson, 1997).

Psychological DSME interventions focus on the therapeutic alliance between the therapist and the person with diabetes to improve their bio‐psychosocial outcomes (Smith, 2012). We classified psychological interventions into the common psychotherapeutic models used in healthcare settings: (a) supportive or counselling therapy (Rogers, 1976); (b) cognitive behaviour therapy (Beck, 1976); (c) brief psychodynamic psychotherapy (Malan, 1963); and (d) interpersonal therapy (Klerman, Weissman, Rounsaville, & Chevron, 1984). Studies where the model of the intervention was unclear were included if they used one or more psychological techniques that could be coded into the above classification. Techniques such as relaxation, activity scheduling, problem‐solving, goal setting, contract setting, cognitive restructuring and stress management were classified as cognitive behaviour therapy (CBT). Techniques such as motivational interviewing and non‐directive counselling were classified under counselling therapy. We reported only one analysis for studies with several intervention groups with the most intensive intervention as the experimental one. Intensity was defined by approach (most intensive was group based rather than individual intervention), type of DSME intervention (most intense was psychological followed by educational), number of sessions and duration of the intervention.

### 3.3 Data extraction

The first reviewer (AM) screened all titles from the searches to exclude studies that were irrelevant. Following this, three reviewers (AM, KW and ES) independently screened the title and abstracts using an eligibility checklist. The full texts of the potentially eligible studies were retrieved for full review and final selection. Studies written in a language other than English were translated by native speakers: a nurse practitioner (Chinese articles) and a pharmacist (Japanese article). Data for the studies were extracted by the first reviewer and verified by the second and third reviewers, (KW and ES) and finally, the fourth reviewer Khalida Ismail (KI) for accuracy and completeness. Any discrepancies in the extracted data were discussed by all 4 reviewers for a 100% consensus.

Data were extracted based on the following: (a) general information (author, title, citation and country); (b) study characteristics (study design, number of participants at baseline and follow‐up, clinical subgroups, demographic details); (c) intervention and setting (setting where intervention delivered and description of it); and (d) outcome data (baseline and follow‐up measure).

### 3.4 Quality assessment

The quality of the studies was assessed using the Jadad score (Jadad et al., 1996) by the first and second reviewers. The quality of included studies was assessed according to 3 appraisal elements: (a) selection bias (randomization procedure and allocation concealment); (b) blinding (masking of outcome assessor but not participants and therapist because DSME intervention cannot be concealed); and (c) attrition bias (withdrawals or dropouts). Studies were then scored on a scale between 0–5. Studies scoring greater than 3 demonstrated high quality.

### 3.5 Data synthesis and analysis

Overview and characteristics of included studies are presented in summary table (Table 2). Meta‐analysis was not conducted due to the heterogeneity of the intervention programmes, populations and outcome measurement. The primary outcome was improvement in glycaemic control (HbA1c, % or mmol/mol). Cohen's d effect size of HbA1c results was used to measure the magnitude of the difference in the outcome between the intervention and control groups, where a value of 0.2 represents a small effect size, 0.5 represents a moderate effect size, and 0.8 represents a large effect size (Cohen, 1988). The secondary outcomes were other metabolic control measures, such as body mass index (BMI) (kg/m^2^), cholesterol level (mmol/L), fasting plasma glucose level (mmol/L) and blood pressure (mmHg), as well as psychosocial variables such as self‐reported quality of life, self‐efficacy and level of depression.

**Table 2:** Overview of the eligible studies examining the effects of Diabetes Self‐Management Education (DSME) interventions for people with type 2 diabetes mellitus (T2DM) in the Asian Western Pacific (AWP) region

First author/Country/Type of study/Year	Number of participants recruited/at follow‐up	Clinical subgroup	Mean age (*SD* or range), years	Type and duration of intervention (intervention group)	Regimen in intervention group and speciality of therapist	Type and duration of intervention (control group)	Regimen in control group and speciality of therapist	Effect size of HbA1c (d)	Other outcomes (intervention vs. control)	Follow‐up (months)	Setting (community vs. clinical)	Quality (Jadad Score)
Campbell/Australia/RCT/1996 (Campbell et al., 1996)	33/19	<5 years’ duration of T2DM	59 (1.4)	Educational (didactic and facilitative teaching) for 12 months	12 monthly individual educational sessions + quarterly group‐based education (lectures on diabetes self‐management & practical sessions on food selection) by RN and MDT (dietitian, occupational therapist and podiatrist)	Educational (didactic teaching) for 12 months	2 hr of individual educational sessions by RN and dietitian	0.6	↑ Diabetes knowledge score: *p* = 0.361 (ND)	12	Both	Low
Chao/China/RCT/2015 (Chao et al., 2015)	100/100	Elderly (age NS)	69 (6.4)	Educational (didactic and facilitative teaching) for 18 months	18 monthly group educational sessions (lectures and a tailored exercise programme) by manager and health service centre manager; speciality NS	Usual care for 18 months	Usual care; regimen and speciality NS	NR	↑ Diabetes knowledge score: *p* < 0.0001 ↑ Psychological health status: *p* = 0.034 ↑ Healthy diet: *p* = 0.012 ↑Physical activity: *p* = 0.013 ↑ SMBG: *p* = 0.004	18	Clinical	High
Guo/China/RCT/2014 (Guo et al., 2014)	1511/1289	HbA1c: >7.5% + 2 or more OADs	57 (10.4)	Educational (didactic and facilitative teaching) for 4 months	6‐group educational sessions at weeks 0,2,4, 8,12 & 16 (7 topics on self‐management) + 3 telephone follow‐ups at weeks 1,3 & 6 by RN	Educational (didactic teaching) for 4 months	6‐group educational sessions (lectures on self‐management) by RN	0.2	↑ SMBG: *p* < 0.05 (ND) ↑C‐DMSES: *p* = 0.0001 ↑ SDSCA: *p* < 0.001 ↑ MMAS: *p* = 0.0002	4	Clinical	High
Jaipakdee/Thailand/Cluster RCT/2015 (Jaipakdee et al., 2015)	403/384/378	HbA1c: ≥ 7% within 2 months before programme	61.3 (9.7)	Educational (didactic and facilitative teaching) for 6 months with psychological support	6 monthly sessions for 3 hr (diabetes education and skill learning (step‐by‐step) with psychological support called 5C intervention (constructing a problem definition; collaborative goal setting; collaborative problem‐solving; contracting for change; continuing support) by trained nurses and healthcare professionals	Usual healthcare over 6m	Physical examination, monitoring of blood sugar levels, individual health education and consultation from a Registered Nurse and/or other healthcare provider	0.2	↓HbA1c: *p* = 0.334 (NS) ↓FPG: *p* = 0.001 ↑Health behaviour score: *p* < 0.001 ↓Weight: *p* = 0.001 ↓PHQ−9: *p* = 0.495 (NS) ↑QOL: *p* < 0.001	3 & 6	Clinical	High
Krass/Australia/RCT/2007 (Krass et al., 2007)	335/289	HBA1c: ≥7.5% + 1 OAD/ insulin; Hba1c: ≥7.0% + 1 OAD or insulin/1 AHT/angina or lipid‐lowering drug	62 (11.0)	Educational (didactic and facilitative teaching) for 6 months	5 individual educational sessions on self‐management by pharmacist + daily self‐monitoring blood glucose level	Educational (didactic teaching) for 6 months	2 individual educational sessions (at beginning and end of the intervention) by pharmacist	0.1	↓ BMI: *p* = 0.37 (ND) ↑ QOL (EQ−5D): *p* = 0.07 (ND)	6	Community	High
Li/China/ RCT/2012 (Li et al., 2012)	280/248	NS	65 (12.2)	Educational (didactic and facilitative teaching) for 18 months	12 monthly health educational club (educational session for 2 hr on self‐management) + 12 telephone follow‐ups (twice monthly) for 6 months + quarterly outdoor activity; speciality NS	NS	Regimen and speciality NS	0.5	↓ FPG: *p* = 0.004 ↓post‐prandial glucose: *p* = 0.003 ↓ HbA1c: *p* = 0.004	18	Community	High
Liu/China/RCT/2012 (Liu et al., 2012)	233/176	NS	62 (9.8)	Psychological (CBT) for 12 months	12 monthly group visit sessions (2.5 hr of each sessions includes lectures, group discussion, action plan) by general practice team (one GP, one physician and one RN)	Usual care for 12 months	Usual care by GP	NR	↑ Diabetes Self‐Efficacy Scale (Stanford Patient Education Research Centre): *p* = 0.02 ↑ Physical activity: *p* = 0.0001 ↑ Depression: *p* = 0.43	12	Community	High
Moriyama/Japan/RCT/2009 (Moriyama et al., 2009)	75/65	NS	66 (8.9)	Psychological (CBT & counselling therapy using motivational interviewing) underpinned by transtheoretical model for 12 months	1 pre‐readiness assessment (transtheoretical model) + 12 monthly individual educational sessions (interview using motivational interviewing) each session lasts for 30 min on self‐management + 6 telephone follow‐ups every fortnight + 1 educational session for carer + 12 monthly individual goal setting by RN	Usual care for 12 months	Usual care + written educational materials on clinical characteristics, treatment available & self‐management measures	0.1	↑ QOL (WHO‐QOL26): *p* = 0.005 ↑Self‐Efficacy: *p* = 0.0001 ↑Physical activity: *p* = 0.520 (ND) ↑Lose weight: *p* = 0.004 ↑ Healthy diet: 0.046	12	Clinical	High
Ng/Singapore/Non‐RCT/2014 (Ng & Sim, 2014)	50	Newly diagnosed duration NS	NR	Educational (didactic teaching) underpinned by self‐efficacy theory for 3 months	Group educational session and regimen; speciality NS	Educational (didactic teaching) for 3 months	Individual educational sessions; regimen and speciality NS	0.1	↑ Physical activity: *p* > 0.05 (ND) ↑ Self‐foot assessment: *p* = 0.984 (ND) ↑ Healthy diet: *p* > 0.05 (ND) ↑ Quit Smoking: *p* > 0.05 (ND)	3	Clinical	Low
Roberts/Australia/Retrospective cohort study/2017 (Roberts et al., 2017)	219	NR	62 (12)	Educational (didactic teaching) for 12 months	1 hr of clinical assessment + 6 weekly group education each session last for 2 hr + proactive recalls at 3, 6, and 12 months by allied health professionals (dietitian, podiatrist) led by the diabetes nurse educator	Educational (didactic teaching) for 12 months	1 hr of clinical assessment + attended at least 1‐group educational session (2 hr) + proactive recalls at 3, 6, and 12 months by same speciality in the intervention group	0.33	↑ Cholesterol: *p* < 0.001 ↑ BMI: *p* = 0.003 ↑QOL: *p* < 0.001 ↑Psychological distress: *p* = 0.016 ↑HbA1c: *p* = 0.134 (NS)	12	Community	Low
Shi/China/RCT/2010 (Shi et al., 2010)	157	Newly diagnosed ≤ 12 months at recruitment period	46 (6.9)	Psychological (counselling therapy) underpinned by self‐efficacy theory for 1 month	4 weekly group educational sessions for 2 hr (counselling on diet & exercise, peer role model for SMBG, persuasion & reinforcement strategies to eliminate barriers) + 2 weekly telephone counselling sessions for 5–15 min (month 4) by RN	Usual care for 4 months	Treatment as usual; regimen and speciality NS	NR	↑ DMSES: *p* = 0.0001 ↑ SDSCA: *p* = 0.0001	4	Clinical	High
Shibayama/Japan/RCT/2007 (Shibayama et al., 2007)	148/134	HbA1c: 6.5−8.5%	62 (7.5)	Psychological (CBT) for 12 months	12 monthly individual counselling sessions for 25 min (self‐management and stress management) by certified expert nurse	Usual care for 12 months	Usual monthly follow‐up by physician	0.2	↑ QOL (SF−36): *p* > 0.05 (ND) ↑ PAID: *p* = 0.57(ND)	12	Clinical	Low
Sone/Japan/RCT/2010 (Sone et al., 2010)	2033/1304	HbA1c: ≥ 6.5%	59 (6.9)	Educational (didactic and facilitative teaching) for 12 months	Self‐management written educational materials + 12 self‐managements individual education (10 min’ additional session from control group) during routine follow‐up by MDT (physician, RN, dietitian) + fortnightly telephone follow‐up by MDT (RN, dietitian & clinical psychologist) + progress diary and pedometer	Educational (didactic teaching) for 12 months	Written educational materials and usual routine follow‐up by physician; regimen NS	0.1	↑ Low fat diet: *p* = 0.30 (ND) ↑ Physical activity: *p* = 0.037	48	Clinical	High
Song/Korea/Non‐RCT/2012 (Song et al., 2012)	40/37	Elderly (age NS)	71 (4.8)	Educational (didactic and facilitative teaching) for 3 months	12 weekly group educational sessions (lectures on self‐management) for 1 hr + 24 biweekly exercise classes for 2 hr + one‐to‐one counselling & instruction sessions at the end of the intervention by a RN and 2 assistants; speciality NS	Usual care for 3 months	Usual care; regimen NS	0.5	↑ DSMB: *p* = 0.006 ↑ FPG: *p* = 0.263 ↑ Total CHO: *p* = 0.782 ↑ Triglyceride: *p* = 0.021 ↑HDL‐C: *p* = 0.024 ↑LDL‐C: *p* = 0.976 ↑body weight (kg): *p* < 0.001 ↑BMI: *p* < 0.001	3	Community	Low
Sun/China/RCT/2008 (Sun et al., 2008)	150	Overweight with BMI: ≥23kgm^3^	51 (1.0)	Educational (didactic and facilitative teaching) for 6 months	6 monthly group educational sessions (lectures on self‐management & healthy eating with meal plans) by nutritionist + 24 weekly self‐monitoring blood glucose follow‐up sessions and diet consultations for 30 min by dietitian and medical evaluation by physician if needed + low glycaemic meal replacement (powdered formula) for breakfast	Educational (didactic teaching) for 6 months	Monthly educational sessions (diet and physical instruction only) by nutritionist	0.6	↑ Low‐carb diet: *p* = 0.634 (NS) ↑ High fibre intake: *p* = 0.010 ↑ Physical activity (PCS): *p* = 0.004 ↑ Mental health (MCS): *p* = 0.017	6	Community	Low
Tan/Malaysia/RCT/2011 (Tan et al., 2011)	164/151	HbA1c: >7.0%	54 (10.3)	Psychological (counselling therapy) underpinned by self‐efficacy theory for 3 months	2 monthly individual educational sessions (self‐management and problem‐solving skills using verbal persuasion, role modelling, physiological state) + 1 telephone follow‐up by RN	Usual care for 3 months	Follow‐up at 3 months by physician	0.5	↑ Diabetes knowledge score: *p* = 0.001 ↑ SMBG: *p* = 0.001 ↑ MMAS: *p* = 0.008 ↑ Low fat diet: *p* > 0.05 (NS) ↑ Physical activity: *p* = 0.001 ↑ BMI: *p* > 0.05 (NS)	3	Clinical	High
Wei/China/RCT/2008 (Wei et al., 2008)	456/338	NS	69 (9.7)	Educational (didactic teaching) for 8 months	8 monthly individual educational sessions (lecture and discussion of diet plans and self‐management activities) by family physician	Usual care for 8 months	Treatment as usual; regimen and speciality NS	NR	↑ FPG: *p* = 0.002 ↑ BMI: *p* = 0.124 (ND) ↑ lose weight: *p* = 0.038	8	Community	Low
Wong/Hong Kong/Observational matched cohort study/2014 (Wong et al., 2014)	2,282	HbA1c: ≥7.0%	65 (10.7)	Psychological (CBT) underpinned self‐efficacy theory for 12 months	Total of 5 hr’ group educational sessions on self‐management (goal setting, problem‐solving, stress management) by healthcare professional; speciality NS	Usual care for 12 months	Received diabetes follow‐up from Hong Kong Hospital Authority GOPC; speciality NS	0.1	↑ Decrease visit to GOPC: *p* = 0.001 ↑ Decrease visit to SOPC: *p* = 0.001 ↑ Decrease visit to ED: *p* = 0.865 (ND) ↑ Decrease inpatient admission: *p* = 0.615(ND)	12	Clinical	Low
Yang/China/Non‐RCT/2007 (Yang et al., 2007)	113	NS	48–71	Educational (didactic teaching) for 6 months	1 introductory educational session by endocrinologist + 12 fortnightly individual Educational sessions by physician	Educational (didactic teaching) for 6 months	1 Introductory educational session + 6 or 12 telephone consultations once every 1 or 2 months by physician	0.4	↑ BMI: *p* > 0.05 (ND)	6	Clinical	Low
Yuan/Hong Kong/RCT/2014 (Yuan et al., 2014)	88/76	> 1‐year duration of T2DM	58 (8.3)	Educational (didactic and facilitative teaching) for 2 months	8 weekly group educational sessions (lectures on self‐management) for 2 hr + self‐management guidance by nutritionist	Usual care for 2 months	Received standard medical nutrition advice; regimen and speciality NS	0.3	↑ Lose weight: *p* = 0.066 (ND) ↑ BMI: *p* = 0.019	2	Community	High
Zhou/China/RCT/2011 (Zhou et al., 2011)	280/248	NS	65 (12.2)	Educational (didactic and facilitative teaching) underpinning self‐efficacy theory for 18 months	12 monthly group educational session for 2 hr (self‐management) + 12 telephone follow‐up (fortnightly) for 6 months + quarterly outdoor activities; speciality NS	NS	Regimen and speciality NS	NR	↑ Self‐anxiety scale: *p* < 0.001 ↑ Self‐rating depression scale: *p* = 0.001 ↑ QOL: *p* < 0.001	18	Community	Low

Abbreviations: ↑, improvement; ↓, worsening; AHT, anti‐hypertensive; BMI, body mass index; BP, blood pressure; CBT, cognitive behaviour therapy; CC, compliance coefficient; C‐DIMES, Chinese version of the Diabetes Self‐efficacy Scale; C‐DMSES, Chinese Diabetes Management Self‐Efficacy Scale; d, Cohen’; DC, distensibility coefficient; DKNA. diabetes knowledge; DMSES, Diabetes Management Self‐Efficacy Scale; DSMB, Diabetes Self‐management Behaviour; ED, emergency department; EQ‐5D, EuroQol‐5 Dimension Questionnaire; GOPC, general outpatient clinic; HbA1c, haemoglobin A1c; heart rate; HR; IMT, intima‐media thickness; MCS, Mental component summary of Short‐Form Health Survey (SF 36); MDT, multidisciplinary team; MMAS, Morisky Medication Adherence Scale; ND, no difference; NR, not reported; NS, not specified; OAD, oral anti‐diabetic agent; PAID, Problem Areas in Diabetes Questionnaire; PAIDS, Problem Areas in Diabetes Scale; PCS, Physical component summary of Short‐Form Health Survey (SF 36); PHQ‐9, Patient Health Questionnaire; PWV, pulse wave velocity; QOL, quality of life; RDSA, Revised Diabetes Self‐care Activities; RN, Registered Nurse; SAS, Self‐Anxiety Scale; SDS, Self‐Rating Depression Scale; SDSCA, Summary of Diabetes Self‐Care Activities; SMBG, self‐monitoring blood glucose; SPOC, specialist outpatient clinic; T2DM, type 2 diabetes mellitus.

## 4 RESULTS

**Figure 1:**
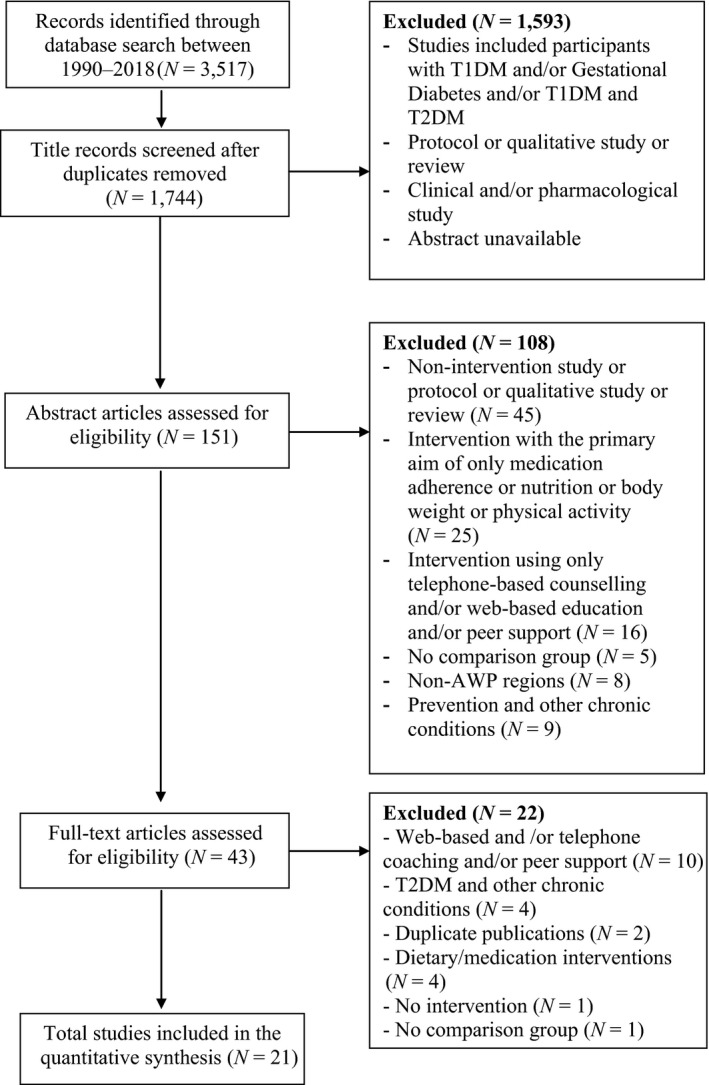
Systematic Review flow diagram

A total of 1,744 non‐duplicated publications were screened, 151 abstracts were assessed for eligibility, and 43 publications required full‐text review before a decision could be made. Twenty‐one studies fulfilled the inclusion criteria, and the search processes are illustrated in Figure 1. The interventions varied considerably according to the number and duration of sessions; however, the content was mostly similar focusing on diabetes self‐management.

### 4.1 Study characteristics

Twenty‐one studies were analysed in this systematic review. Sixteen studies (Campbell, Redman, Moffitt, & Sanson‐Fisher, 1996; Chao et al., 2015; Guo et al., 2014; Jaipakdee, Jiamjarasrangsi, Lohsoonthorn, & Lertmaharit, 2015; Krass et al., 2007; Li et al., 2012; Liu et al., 2012; Moriyama et al., 2009; Shi, Ostwald, & Wang, 2010; Shibayama, Kobayashi, Takano, Kadowaki, & Kazuma, 2007; Sone et al., 2010; Sun et al., 2008; Tan, Magarey, Chee, Lee, & Tan, 2011; Wei et al., 2008; Yuan et al., 2014; Zhou et al., 2011) were RCTs; 4 studies (Ng & Sim, 2014; Song et al., 2012; Wong et al., 2014; Yang et al., 2007) were observational matched cohort studies; and one was a retrospective cohort study (Roberts, Ward, Russell, & O’Sullivan, 2017).

The duration of interventions ranged from 6 weeks–18 months. Eight studies were conducted in China (Chao et al., 2015; Li et al., 2012; Liu et al., 2012; Shi et al., 2010; Sun et al., 2008; Wei et al., 2008; Yang et al., 2007; Zhou et al., 2011), 3 in Japan (Moriyama et al., 2009; Shibayama et al., 2007; Sone et al., 2010), 3 in Australia (Campbell et al., 1996; Krass et al., 2007; Roberts et al., 2017) and 2 in Hong Kong (Wong et al., 2014; Yuan et al., 2014), as well as 1 each in Singapore (Ng & Sim, 2014), Korea (Song et al., 2012), Malaysia (Tan et al., 2011) and Thailand (Jaipakdee et al., 2015). The mean age range of study participants was 45–71 years with most (*N* = 21, 91%) of studies having a mean population age of 55 years and above.

Most studies in the review (*N* = 21, 91%) assessed glycated haemoglobin (HbA1c, %) as the primary outcome with the effect size (Cohen's d) ranging from 0.1–0.6 and psychosocial well‐being or quality of life as the secondary outcome. Two studies were translated into English from the original Chinese article (Li et al., 2012; Zhou et al., 2011). With regard to quality, 11 studies (Chao et al., 2015; Guo et al., 2014; Jaipakdee et al., 2015; Krass et al., 2007; Li et al., 2012; Liu et al., 2012; Moriyama et al., 2009; Shi et al., 2010; Sone et al., 2010; Tan et al., 2011; Yuan et al., 2014) were classified as “high”. Ten studies as “low” including five RCTs (Campbell et al., 1996; Shibayama et al., 2007; Sun et al., 2008; Wei et al., 2008; Zhou et al., 2011), two non‐RCTs (Song et al., 2012; Wong et al., 2014), one retrospective cohort study (Roberts et al., 2017) and two were of abstracts only (Ng & Sim, 2014; Yang et al., 2007).

### 4.2 Intervention characteristics

Fourteen studies (Campbell et al., 1996; Chao et al., 2015; Guo et al., 2014; Jaipakdee et al., 2015; Liu et al., 2012; Ng & Sim, 2014; Roberts et al., 2017; Shi et al., 2010; Song et al., 2012; Sun et al., 2008; Wong et al., 2014; Yuan et al., 2014; Zhou et al., 2011) were group‐based interventions, while 7 studies (Krass et al., 2007; Moriyama et al., 2009; Shibayama et al., 2007; Sone et al., 2010; Tan et al., 2011; Wei et al., 2008; Yang et al., 2007) used an individual approach.

Of the 21 studies, there were several clinical subgroups among the populations studied: (a) 2 studies (10%) were conducted among elderly people; (b) 4 (19%) included people with less than 5 years duration of T2DM; (c) 1 (5%) was conducted among overweight people with T2DM (BMI > 23kgm^3^); (d) 7 (33%) were implemented among people with T2DM with sub‐optimal diabetes control; and the remaining 7 (33%) did not specify a clinical sub‐group.

Fifteen interventions were classified as predominantly educational with 11 using a combination of didactic and facilitative teaching (Campbell et al., 1996; Chao et al., 2015; Guo et al., 2014; Jaipakdee et al., 2015; Krass et al., 2007; G. Li et al., 2008; Sone et al., 2010; Song et al., 2012; Sun et al., 2008; Yuan et al., 2014; Zhou et al., 2011) and 4 using didactic teaching alone (Ng & Sim, 2014; Roberts et al., 2017; Wei et al., 2008; Yang et al., 2007). Six studies were grouped as predominantly psychological with only one study explicitly stating that the intervention used CBT and motivational interviewing (Moriyama et al., 2009). The remaining 5 studies used other psychological techniques; 3 used CBT strategies such as problem‐solving and goal setting (Liu et al., 2012; Shibayama et al., 2007; Wong et al., 2014) and 2 studies used counselling therapy (Shi et al., 2010; Tan et al., 2011). None of the psychological intervention group studies used a psychodynamic or interpersonal model of therapy. There were interventions underpinned by various theories; 5 with self‐efficacy theory, 2 of these were educational (Ng & Sim, 2014; Zhou et al., 2011) and 3 were psychological interventions (Shi et al., 2010; Tan et al., 2011; Wong et al., 2014). In the 6 studies applying psychological intervention, one study was underpinned by the transtheoretical theory (Moriyama et al., 2009) and one by behaviour change (Jaipakdee et al., 2015).

### 4.3 Quality assessment

In general, most of the studies included were of reasonable methodological quality and the abstract of the included studies was able to provide adequate information particularly on the aims, methods and findings of each study. Of twenty‐one studies, 12 (60%, *N* = 21) of the reviews scored three and above against the Jadad score, which were then categorized as high in quality. Table 3 illustrates the elements of the Jadad score for the included studies. Six studies provided limited information on the randomization strategies used (Campbell et al., 1996; Shibayama et al., 2007; Sun et al., 2008; Wei et al., 2008; Yang et al., 2007; Zhou et al., 2011).

**Table 3:** Quality of the 21 studies as assessed by the Jadad score

Study	Randomization	Appropriate randomization utilized	Blinding present	Appropriate blinding method utilized	Description of withdrawals and dropouts	Score	Quality (Jadad Score)
Campbell et al., (1996)	1	0	0	0	1	2	Low
Chao et al., (2015)	1	1	0	0	1	3	High
Guo et al., (2014)	1	1	0	0	1	3	High
Jaipakdee et al., (2015)	1	1	0	0	1	3	High
Krass et al., (2007)	1	1	0	0	1	3	High
Li et al., (2012)	1	1	0	0	1	3	High
Liu et al., (2012)	1	1	0	0	1	3	High
Moriyama et al., (2009)	1	1	0	0	1	3	High
Ng and Sim (2014)	0	0	0	0	0	0	Low
Roberts et al., (2017)	0	0	0	0	1	1	Low
Shi et al., (2010)	1	1	0	0	1	3	High
Shibayama et al., (2007)	1	0	0	0	1	2	Low
Sone et al., (2010)	1	1	0	0	1	3	High
Song et al., (2012)	0	0	0	0	1	1	Low
Sun et al., (2008)	1	0	0	0	1	2	Low
Tan et al., (2011)	1	1	0	0	1	3	High
Wei et al., (2008)	1	0	0	0	1	2	Low
Wong et al., (2014)	0	0	0	0	0	0	Low
Yang et al., (2007)	1	0	0	0	1	2	Low
Yuan et al., (2014)	1	1	0	0	1	3	High
Zhou et al., (2011)	1	0	0	0	0	1	Low

It was noted that none of the trials explicitly explained appropriate methods of blinding, although the majority did describe numbers of withdrawals and dropouts. In most of the studies, the data were analysed using the intention‐to‐treat principle, which helps to preserve the sample size, which is an important criterion for statistical power.

For the 15 studies with an educational intervention, the majority of the included studies were RCTs (60%; 10 studies) where the studies were relatively balanced; four studies were implemented in a clinical setting (Chao et al., 2015; Guo et al., 2014; Jaipakdee et al., 2015; Sone et al., 2010) and five studies in the community (Krass et al., 2007; Sun et al., 2008; Wei et al., 2008; Yuan et al., 2014; Zhou et al., 2011). Only one study was implemented in both clinical and community settings (Campbell et al., 1996). Six studies were classified as high quality by the Jadad score (Chao et al., 2015; Guo et al., 2014; Jaipakdee et al., 2015; Krass et al., 2007; Sone et al., 2010; Yuan et al., 2014). Of 6 studies with a psychological intervention, the majority were also RCTs (83%; 5 studies) and conducted in a clinical setting (Moriyama et al., 2009; Shi et al., 2010; Shibayama et al., 2007; Tan et al., 2011), with only one study taking place in the community (Liu et al., 2012). Four included studies were considered high quality by the Jadad score (Liu et al., 2012; Moriyama et al., 2009; Shi et al., 2010; Tan et al., 2011).

Significant scores were observed in the following measures: the 15‐item diabetes knowledge scale (DKNA) (Chao et al., 2015; Tan et al., 2011); SMBG (Chao et al., 2015; Tan et al., 2011); MMAS (Guo et al., 2014); the Diabetes Management Self‐Efficacy Scale (DMSES) (Guo et al., 2014; Shi et al., 2010); and the Summary of Diabetes Self‐Care Activities (SDSCA) (Guo et al., 2014), which were all found in both educational and psychological RCTs implemented in a clinical setting and rated as high quality. Additionally, significant scores were observed on the quality of life (QOL) measures among RCTs using an educational approach in a clinical setting (Jaipakdee et al., 2015) as well as an educational intervention of retrospective cohort studies in a community setting (Roberts et al., 2017; Zhou et al., 2011).

### 4.4 Impact of the educational and psychological interventions on glycaemic control

Sixteen studies examined the HbA1c (%) as an outcome to measure the impact of DSME (educational and psychological) interventions. Eight studies (50%; *N* = 16) reported statistically significant improvements in glycaemic control, for both educational and psychological interventions. Four out of 8 studies (50%; *N* = 8) (Campbell et al., 1996; Li et al., 2012; Song et al., 2012; Sun et al., 2008) were of group‐based interventions, demonstrating moderate effect size ranging from 0.5–0.6. While, six out of seven (85%; *N* = 7) were of individual intervention studies (Krass et al., 2007; Moriyama et al., 2009; Shibayama et al., 2007; Sone et al., 2010; Wei et al., 2008; Yang et al., 2007), having a smaller effect size of HbA1c ranging from 0.1–0.2. Among the studies that yielded moderate (good) effect size, four studies (80%, *N* = 5) (Campbell et al., 1996; G. Li et al., 2008; Song et al., 2012; Sun et al., 2008; Yuan et al., 2014) represented high‐intensity programmes (>10 hr sessions), as defined in the systematic review of RCTs on behavioural interventions (Pillay et al., 2015). In addition, five group‐based intervention studies reported significant improvement in HbA1c and 2 of these (40%; *N* = 5) (Li et al., 2008; Sun et al., 2008) reported moderate effect size.

### 4.5 Impact of the educational and psychological interventions on psychological well‐being, diabetes knowledge and self‐management

Two studies (Campbell et al., 1996; Chao et al., 2015) measured diabetes knowledge using validated questionnaires. These include the DKNA and the Revised Diabetes Self‐care Activities (RDSA) questionnaires modified from the Diabetes Self‐care Activities Questionnaire used in Malaysia. Only one (Chao et al., 2015) demonstrated a significant difference between the intervention and the control group. Six studies (Chao et al., 2015; Guo et al., 2014; Ng & Sim, 2014; Shi et al., 2010; Song et al., 2012; Tan et al., 2011) measured at least one aspect of diabetes self‐management. However, only four studies (Chao et al., 2015; Guo et al., 2014; Song et al., 2012; Tan et al., 2011) demonstrated statistically significant improvement in the intervention group.

Of 6 studies, four studies assessing psychosocial self‐efficacy have shown statistically significant improvement (Guo et al., 2014; Liu et al., 2012; Moriyama et al., 2009; Shi et al., 2010) and only 1 (Moriyama et al., 2009) of these was carried out using an individual approach, while the other three were group based (Guo et al., 2014; Liu et al., 2012; Shi et al., 2010). Five studies measured the quality of life using validated questionnaires (EQ5D, WHO‐QOL26, SF‐36 and Chinese version of DQOL), with three of these reporting statistically significant results: one study used an individual psychological intervention (Moriyama et al., 2009) and 2 studies used group‐based educational (didactic and facilitative) teaching (Jaipakdee et al., 2015; Zhou et al., 2011). However, the other two studies (Krass et al., 2007; Shibayama et al., 2007) reported no difference in the quality of life outcomes and were individual educational and psychological interventions, respectively.

### 4.6 Impact of additional components, setting and speciality of the educators on glycaemic control

There were four studies (Campbell et al., 1996; Chao et al., 2015; Jaipakdee et al., 2015; Song et al., 2012) which integrated practical sessions such as exercise classes and healthy diet preparation into the DSME intervention. Only two of four studies (Campbell et al., 1996; Song et al., 2012) reported a moderate effect size for HbA1c. Five studies (Guo et al., 2014; Liu et al., 2012; Moriyama et al., 2009; Sone et al., 2010) integrated telephone follow‐ups as part of their intervention, and only one reported a moderate effect size of HbA1c (Tan et al., 2011). In addition, three (33%; *N* = 9) studies (Campbell et al., 1996; Song et al., 2012; Sun et al., 2008) were delivered by multidisciplinary teams comprising of diabetes nurse educators, dietitians, podiatrists, general practitioners (GP) or clinical psychologists and achieved acceptable effect sizes (ranging 0.5–0.6) compared with interventions delivered by only one healthcare provider such as a trained nurse educator, physician or nutritionist (11%; *N* = 9) (Tan et al., 2011).

### 4.7 Integration of cultural sensitivity in the interventions

Most of the studies identified by this review have involved the delivery of educational, self‐management and/or psychological interventions for people with T2DM from countries in the AWP region. However, from the examination of the intervention characteristics, none of them has specified specific cultural adaptations to address the needs of local individuals to support diabetes self‐management. The only cultural concessions made and reported were 17 studies mainly conducted in the East and South‐East Asian countries which included the translation of self‐report outcome measurements, such as validated questionnaires and delivery of interventions in native languages (Chao et al., 2015; Guo et al., 2014; Jaipakdee et al., 2015; Liu et al., 2012; Moriyama et al., 2009; Ng & Sim, 2014; Shi et al., 2010; Shibayama et al., 2007; Sone et al., 2010; Song et al., 2012; Sun et al., 2008; Tan et al., 2011; Wei et al., 2008; Wong et al., 2014; Yang et al., 2007; Yuan et al., 2014; Zhou et al., 2011).

## 5 DISCUSSION

It is important that people with T2DM undertake adequate self‐management to optimize blood glucose levels which may reduce and delay diabetes‐related complications (UK Prospective Diabetes Study Group, 1998). Therefore, DSME is necessary to support people with T2DM to develop effective diabetes self‐management skills. DSME is well integrated in Western developed countries, and this review highlights the growing number of programmes being developed in the AWP region and provides some evidence that DSME is effective in improving glycaemic control for people with T2DM living in this area. A total of 21 studies were identified which used interventions with various educational or psychological therapeutic approaches and modes of delivery.

It was found that most group‐based DSME interventions provided a good effect on glycaemic control compared with one‐to‐one interventions, particularly programmes conducted for 10 hr or more (high‐intensity programmes). Interventions that integrated practical sessions reported an exceptional clinical improvement in glycaemic control (moderate effect size Cohen's d > 0.5). There was a trend from 2007 onwards towards multidimensional interventions (involving facilitative teaching and psychological elements) rather than on didactic teaching alone. There is little evidence to recommend a specific theoretical model as the most effective for DSME from the available analysis; however, self‐efficacy theory was widely used. Hence, we have little understanding of how intervention components promote behavioural modification or lifestyle change which may help improve clinical outcomes as only one‐third of our included studies measured at least one aspect of diabetes self‐management. Despite the small proportion of studies, they generally reported positive effects for glycaemic control.

No direct comparison can be made between the present systematic review and any other review conducted in the AWP region as there are none. However, the results from this review are consistent with the review by Chrvala, Sherr, and Lipman (2016) who reported that a combination of group and active participation in DSME improve diabetes management and outcomes in Western countries. This review is also consistent with the review by Steed, Cooke, and Newman (2003) who reported that didactic teaching approaches alone had less overall effect on glycaemic control and Norris, Engelgau, and Narayan (2001) who concluded that interventions incorporating “hands‐on” sessions were more effective than didactic approaches. The finding that high‐intensity programmes appeared to be more beneficial supports the review by Pillay et al. (2015) who revealed that DSME with less than 10 hr of sessions (less intensive) is less effective compared with more intensive sessions. Another systematic review and meta‐analysis by Steinsbekk, Rygg, Lisulo, Rise, and Fretheim (2012) synthesizing DSME RCTs concluded that interventions conducted with longer hours (more than 12 hr and between 6–10 sessions) have proven to be more beneficial in optimizing blood glucose level.

It is worth noting that multidisciplinary teams of DSME which involve more than one member of health professionals may contribute to effectiveness, though this has yet to be confirmed in RCTs and observational studies according to a review by Chrvala et al. (2016). It was unclear what the specific cultural elements of the included studies are, but all of the non‐English speaking countries implemented the DSME interventions in their mother tongue and the questionnaires used to evaluate the programmes were translated and validated. Previous systematic reviews of culturally tailored DSME conducted among minority ethnics in Western nations reported a positive impact on behaviour change and glycaemic control when it is linguistically acceptable (Pillay et al., 2015).

### 5.1 Limitations

There is a possibility of publication bias as only published data were included. Fourteen studies (64%) included in the analysis had less than 12 months of follow‐up data; therefore, long‐term outcomes could not be assessed. In addition, the assessment of psychosocial outcomes in all studies was based on self‐report questionnaires which may introduce some bias. This review included non‐randomized study designs and was carried out using a discursive analysis rather than meta‐analysis, due to the heterogeneity in both the interventions and outcome measures, as well as the difference in populations and settings. In terms of outcomes, this review mainly focuses on glycaemic control (HbA1c), due to the issue of extracting or calculating the effect size when the secondary outcomes were made using different measures, such as for diabetes knowledge, quality of life, self‐efficacy, adherence and physical activity. Positive psychology is a relatively new approach that has been increasingly used as a promising technique to promote health; however, we did not encounter this approach in our search. In future searches, it might be useful to define this in the search strategy to see whether this changes the inclusion of positive psychology approaches.

Despite the Jadad scoring system being simple and easy to use with known reliability and external validity, there are some flaws in the scale. It should be noted that the scoring system does not address the appropriateness of data analysis or allocation concealment (which is one of the parameters to avoid bias in research), or the assessment of intention‐to‐treat analysis. It also focuses on blinding, which is challenging in RCTs with complex interventions.

Although blinding is gold standard in research design, blinding in most complex intervention RCTs is often not feasible. Complex intervention research such as DSME intervention trials is mainly conducted to determine effectiveness rather than efficacy in the traditional RCTs and often measure complex outcomes as well as consisting of multiple interactive elements that make it challenging to blind (Mustafa, 2017). Although randomization was implemented, there is still a potential of confounding bias, either consciously or unconsciously, which may have a negative impact on the integrity of the complex intervention trials.

## 6 CONCLUSION

This review identified and summarized the available evidence in the AWP region from the 21 studies regarding the effectiveness of DSME to improve diabetes self‐management. The results suggest that overall group‐based DSME is associated with improved clinical and psychosocial outcomes and interventions underpinned by behavioural theory with longer contact hours and the inclusion of active, hands‐on participatory sessions may maximize the potential benefit of these programmes. Likewise, involving the participation of the multidisciplinary team may also be important. However, what we do not yet know is how to target DSME in this region so that it is culturally appropriate and whether beliefs and attitudes towards diabetes in ethnically diverse AWP communities are being addressed and how this is achieved, which suggests more research is needed. We can conclude that standardized DSME programmes or specific guidelines in this region were limited; therefore, there is an urgent need to develop DSME and make it accessible to people with T2DM among AWP countries.

## 7 RELEVANCE TO CLINICAL PRACTICE

This review may guide healthcare providers or policymakers in designing future culturally tailored programmes for people with T2DM in the AWP region. Successful programmes are likely to be group based, include active participation and longer contact hours, but more research is needed to determine how to address specific cultural beliefs and attitudes towards diabetes. For example, the small feast culture appears to be one of the biggest barriers to maintaining the desired glucose level in Asian cultures. This happens infrequently in Western cultures, where meals are only offered to close friends and family (Douglas, 1975). Our research aligned with an ethnographic study among Middle Eastern people in the United Arab Emirates (UAE) which demonstrated that providing meals to strangers, guests and friends is a way to minimize the social gap (Baglar, 2013). It is considered polite to finish food served to you in Asian cultures and this often hinders efforts at dietary modification. Therefore, the following should be considered when conducting future research: (a) interventions to be tested using randomized trials; (b) interventions incorporating “hands‐on” sessions and psychological techniques, problem‐solving and goal setting; (c) additional outcome measurements of behavioural change and coping skills; (d) explicit use of culturally relevant materials; and (e) and cost‐effectiveness analysis.

## CONFLICT OF INTEREST

None.
